# Effects of statin therapy on clinical outcomes after acute myocardial infarction in patients with advanced renal dysfunction: A propensity score-matched analysis

**DOI:** 10.1371/journal.pone.0183059

**Published:** 2017-08-14

**Authors:** Jin Sug Kim, Weon Kim, Ji Yoon Park, Jong Shin Woo, Tae Won Lee, Chun Gyoo Ihm, Yang Gyun Kim, Ju-Young Moon, Sang Ho Lee, Myung Ho Jeong, Kyung Hwan Jeong, for the Korea Acute Myocardial Infarction Registry Investigators

**Affiliations:** 1 Division of Nephrology, Department of Internal Medicine, Kyung Hee University School of Medicine, Seoul, Republic of Korea; 2 Division of Cardiology, Department of Internal Medicine, Kyung Hee University School of Medicine, Seoul, Republic of Korea; 3 Division of Cardiology, Department of Medicine, Chonnam National University Hospital, Gwangju, Republic of Korea; University of Tampere, FINLAND

## Abstract

**Objective:**

Lipid lowering therapy is widely used for the prevention of cardiovascular complications after acute myocardial infarction (AMI). However, some studies show that this benefit is uncertain in patients with renal dysfunction, and the role of statins is based on the severity of renal dysfunction. In this study, we investigated the impact of statin therapy on major adverse cardiac events (MACEs) and all-cause mortality in patients with advanced renal dysfunction undergoing percutaneous coronary intervention (PCI) after AMI.

**Methods:**

This study was based on the Korea Acute Myocardial Infarction Registry database. We included 861 patients with advanced renal dysfunction from among 33,205 patients who underwent PCI after AMI between November 2005 and July 2012. Patients were divided into two groups: a statin group (n = 537) and a no-statin group (n = 324). We investigated the 12-month MACEs (cardiac death, myocardial infarction, repeated PCI or coronary artery bypass grafting) and all-cause mortality of each group. Subsequently, a propensity score-matched analysis was performed.

**Results:**

In the total population studied, no significant differences were observed between the two groups with respect to the rate of recurrent MI, repeated PCI, coronary artery bypass grafting (CABG), or all-cause mortality. However, the cardiac death rate was significantly lower in the statin group (p = 0.009). Propensity score-matched analysis yielded 274 pairs demonstrating, results similar to those obtained from the total population. However, there was no significant difference in the cardiac death rate in the propensity score-matched population (p = 0.103). Cox-regression analysis revealed only left ventricular ejection fraction to be an independent predictor of 12-month MACEs (Hazard ratio [HR] of 0.979, 95% confidence interval [CI], 0962–0.996, p = 0.018).

**Conclusions:**

Statin therapy was not significantly associated with a reduction in the 12-month MACEs or all-cause mortality in patients with advanced renal dysfunction undergoing PCI after AMI.

## Introduction

Lipid lowering therapy is essential for prevention of major cardiovascular complications in patients diagnosed with cardiovascular disease (CVD), as well as in individuals at high cardiovascular risk [1, 2]. Although chronic kidney disease (CKD) is acknowledged as an independent risk factor for CVD [3], the benefit of lipid lowering therapy in CKD patients remains unclear and debatable. Several meta-analyses demonstrate that the effect of statin therapy is significantly modified by renal function [4, 5]. Recently, large-scale trials have investigated the clinical effect of statins in patients with CKD [6–8]. These trials proposed differing results of the effects of statins, depending on the severity of renal dysfunction. Two studies—the Deutsche Diabetes Dialyse Studie (4D), and A Study to Evaluate the Use of Rosuvastatin in Subjects on Regular Hemodialysis: An Assessment of Survival and Cardiovascular Events (AURORA) trials—which examined patients receiving hemodialysis, reported that statins were not beneficial in preventing cardiovascular complications [6, 7]. However, the Study of Heart and Renal Protection (SHARP) trial showed evidence of their clinical efficacy in a wide range of patients with CKD [8].

Although statins demonstrate a clinical effect on the secondary prevention of cardiovascular events, the above-mentioned trials did not focus on this benefit. Few studies have described the secondary preventive effects of statins after percutaneous coronary intervention (PCI) in patients with renal dysfunction [9–11]. However, most of these studies were performed on patients with mild renal dysfunction (estimated glomerular filtration rate [eGFR] ≤ 60 mL/min/1.73 m^2^ and < 55.9 mL/min/1.73 m^2^) [9, 11]. An increasing body of evidence demonstrates that the pathophysiology of CVD differs based on the severity of renal dysfunction [12, 13]. Additionally, some studies report that as renal function declines, the effectiveness of lipid lowering therapy in prevention of CVD also decreases [12, 14]. Thus, further studies need to be performed in patients with advanced renal dysfunction (eGFR < 30 mL/min/1.73 m^2^).

In this study, we evaluated the clinical impact of statins on major adverse cardiac events (MACEs) and all-cause mortality in patients with advanced renal dysfunction who had undergone PCI after acute myocardial infarction (AMI). Propensity score-matched analysis was performed to minimize the effect of a selection bias occurring due to the effect of potential confounders.

## Methods

### Study population and design

**Fig 1:**
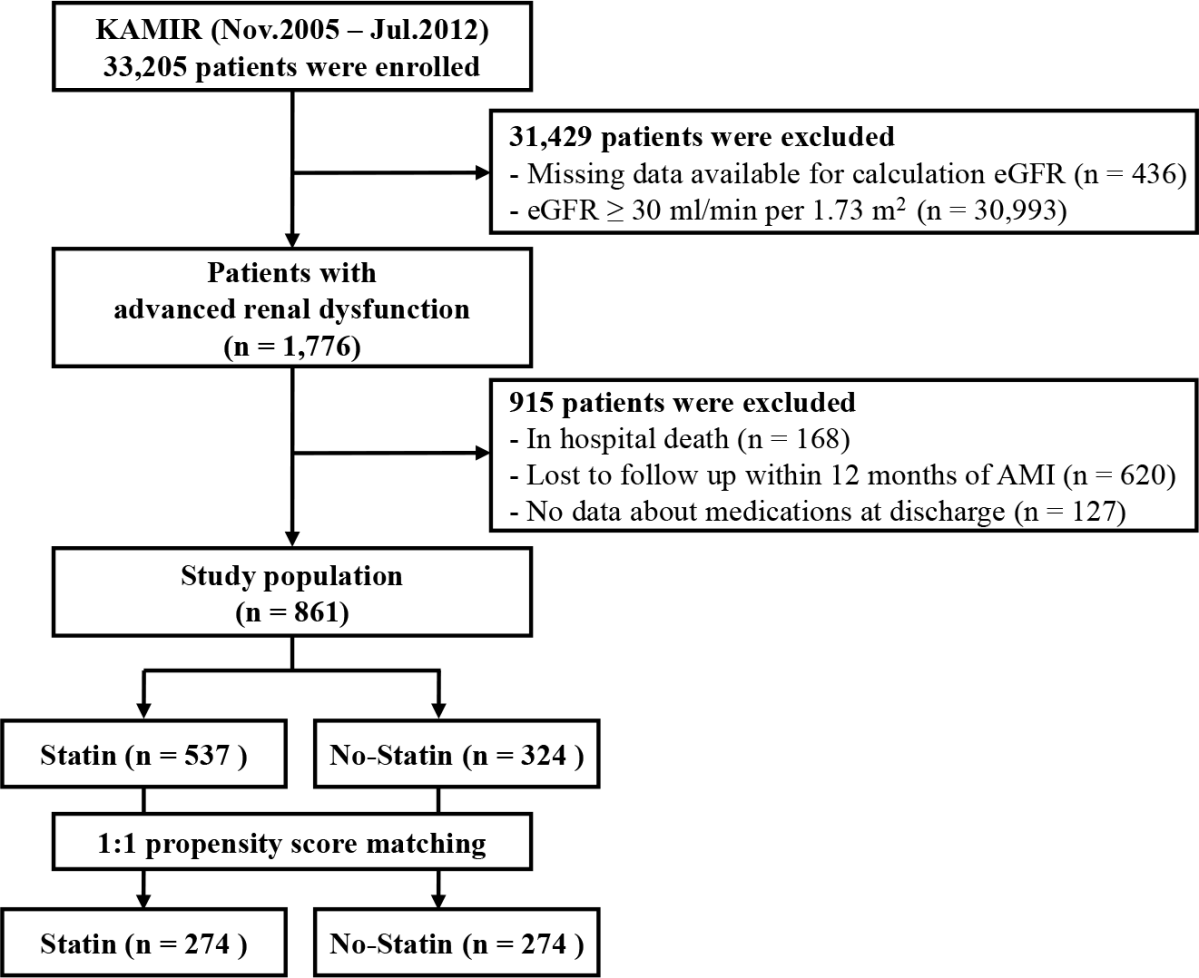
Study flow chart. KAMIR, Korea Acute Myocardial Infarction Registry; eGFR, estimated glomerular filtration rate; AMI, acute myocardial infarction.

Our study population was selected from the Korea Acute Myocardial Infarction Registry (KAMIR) database. Established in November 2005, and supported by the Korean Society of Cardiology, KAMIR is a prospective, observational, multicenter, web-based registry that includes AMI patients in Korea. The registry includes 53 centers with facilities for primary PCI. All data were accessed anonymously for analysis. Between November 2005 and July 2012, 33,205 patients diagnosed with AMI were enrolled in the KAMIR database. As shown in Fig 1, 1,776 patients diagnosed with advanced renal dysfunction, defined as eGFR less than 30 mL/min/1.73 m^2^, were eligible to be included in our study. We excluded patients with missing data regarding medications, in-hospital deaths, or those who were lost to follow-up within 12 months of being diagnosed with an AMI. Finally, 861 patients were enrolled in this study. Patients were divided into two groups (statin vs. no-statin group) based on whether they were prescribed statins as a discharge medication. Additionally, we performed a propensity score-matched analysis for treatment with statins. Details relating to propensity score-matched analysis are described below. The present study was performed based on the ethical guidelines of the 1975 Declaration of Helsinki, as revised in 2000. The study protocol was approved by the Institutional Review Board of all centers, and the approval number was 05–49 obtained from Chonnam National University Hospital. Written informed consent was obtained from all patients. The participating sites are listed in an appendix.

### Definition and variables

A serum creatinine level was measured prior to PCI, and renal function was assessed based on eGFR expressed as mL/min/1.73 m^2^ using the Chronic Kidney Disease Epidemiology Collaboration (CKD-EPI) equation [15]. Advanced renal dysfunction was defined as an eGFR < 30 mL/min/1.73 m^2^. Baseline variables included age, gender, initial blood pressure, body mass index, and several cardiovascular risk factors such as hypertension, diabetes mellitus, dyslipidemia, and previous coronary artery disease (CAD). Laboratory findings including serum lipid profile and cardiac markers, and medications prescribed at discharge, were recorded.

### Study outcomes

The primary study outcome was 12-month MACEs, which included cardiac death, recurrent myocardial infarction (MI), repeated PCI (target lesion or target vessel revascularization, or non-target vessel revascularization), and coronary artery bypass grafting (CABG). Cardiac death was defined as death attributable to arrhythmia or mechanical complications such as free wall rupture and ventricular septal rupture. Recurrent MI was defined as recurrent clinical features with new electrocardiographic findings that were compatible with MI, or increased levels of cardiac markers, these values being at least twice the upper limit of normal. We did not include planned revascularization as the primary study outcome. Secondary study outcome was an all-cause mortality during the 12-month follow-up. All-cause mortality included both cardiac and non-cardiac deaths.

### Statistical analysis

Continuous variables are presented as means ± standard deviations (SDs), and categorical data are reported as frequencies and percentages. Analysis of continuous variables was performed using the Student’s t-test, and categorical data was analyzed using a Chi-square test or the Fisher’s exact test. Survival curves were estimated by the Kaplan–Meier method and compared with the log-rank test. Multiple Cox proportional regression analysis determined the association of variables with 12-month MACEs, after adjusting for several confounders. The results are presented as hazard ratios (HRs) ± 95% confidence intervals (CI), and statistical significance is indicated. To reduce a potential selection bias and to balance differences between the two groups, a propensity score was estimated using a multivariable logistic regression model. Variables included in the logistic regression model used to deduce the propensity score were: age, gender, hypertension, dyslipidemia, Killip class, total cholesterol, low-density lipoprotein (LDL) cholesterol, N-terminal prohormone of brain natriuretic peptide, and discharge medications. The C-statistic for the propensity score derivation was 0.76. Statin and no-statin groups were subsequently matched using a 1:1 matching algorithm on the propensity scores. All statistical analyses were carried out using SPSS software version 19.0 (SPSS Inc., Chicago, IL, USA). A p value < 0.05 was considered statistically significant.

## Results

### Baseline characteristics, laboratory findings, and medications

Baseline characteristics, initial laboratory findings, and medications at discharge are presented in Table 1. Among the 861 patients, 537 (62.4%) patients were treated with statins. The mean age of the study population was 69.4 years (age range: 18–100) and 477 (55.4%) were men. The statin group showed a higher prevalence of hypertension and dyslipidemia compared to the no-statin group. Patients belonging to the no-statin group had a poorer Killip class. Laboratory evaluation revealed that serum total cholesterol and LDL cholesterol values were significantly higher in the statin group. There were some significant differences between the two groups in terms of medications prescribed at discharge. After 1:1 propensity score-matching, 548 patients were matched. In the propensity score-matched populations, no significant differences were observed between the two groups with respect to baseline characteristics, laboratory findings, and medications.

**Table 1:** Baseline characteristics of the study population.

	Total population		p	Propensity-matched population		p
	Statin (n = 537)	No-Statin (n = 324)		Statin (n = 274)	No-Statin (n = 274)	
Age	69.3 ± 11.3	69.6 ± 10.6	0.733	69.8 ± 10.7	69.7 ± 10.6	0.842
Gender (male)	296 (55.1%)	181 (55.9%)	0.832	145 (52.9%)	150 (54.7%)	0.668
Body mass index (Kg/m^2^)	23.4 ± 3.4	23.0 ± 3.17	0.133	23.3 ± 3.1	23.0 ± 3.2	0.399
Systolic blood pressure (mm Hg)	133.7 ± 34.8	131.2 ± 33.9	0.318	135.1 ± 36.3	131.9 ± 34.8	0.314
Diastolic blood pressure (mm Hg)	78.1 ± 18.4	77.4 ± 18.9	0.567	78.6 ± 17.7	77.5 ± 19.2	0.505
Heart rate	82.6 ± 22.6	82.5 ± 22.6	0.944	84.3 ± 21.6	83.1 ± 22.4	0.531
Killip class (more than II)	288 (53.6%)	203 (62.7%)	0.010	167 (60.9%)	166 (60.6%)	0.930
**Cardiovascular risk factors**						
Hypertension	439 (81.9%)	246 (75.9%)	0.035	214 (78.1%)	210 (76.6%)	0.683
Diabetes mellitus	310 (57.8%)	187 (57.7%)	0.973	158 (57.5%)	157 (57.7%)	0.971
Dyslipidemia	94 (17.6%)	27 (8.4%)	< 0.001	23 (8.4%)	20 (7.3%)	0.634
Current smoking	165 (31.4%)	97 (30.2%)	0.712	91 (33.7%)	85 (31.1%)	0.523
Previous CAD	165 (31.0%)	90 (27.9%)	0.329	80 (29.2%)	76 (27.8%)	0.725
**Laboratory findings**						
LVEF (%)	48.04 ± 12.8	46.7 ± 13.1	0.168	47.2 ± 13.1	47.2 ± 12.9	0.952
Glucose (mg/dl)	210.1 ± 119.7	205.9 ± 118.4	0.631	223.4 ± 129.6	209.0 ± 122.9	0.185
Creatinine (mg/dl)	4.7 ± 4.2	4.6 ± 3.7	0.901	4.7 ± 4.2	4.6 ± 3.4	0.657
Estimated GFR (ml/min/1.73 m^2^)	17.1 ± 8.8	16.6 ± 8.8	0.451	17.0 ± 8.8	16.7 ± 8.7	0.693
Maximal CK-MB (U/L)	72.4 ± 119.1	66.9 ± 125.7	0.526	72.2 ± 122.6	67.9 ± 129.2	0.689
Maximal TnI (ng/ml)	31.7 ± 57.6	28.9 ± 74.8	0.566	33.4 ± 65.6	28.0 ± 73.7	0.388
Total cholesterol (mg/dl)	171.1 ± 49.8	158.9 ± 43.2	< 0.001	160.2 ± 41.6	158.5 ± 43.3	0.632
Triglyceride (mg/dl)	127.5 ± 80.4	119.5 ± 74.6	0.148	121.0 ± 85.7	118.9 ± 75.3	0.757
HDL cholesterol (mg/dl)	40.3 ± 12.2	39.7 ± 12.3	0.514	40.5 ± 12.4	39.7 ± 12.4	0.457
LDL cholesterol (mg/dl)	103.2 ± 43.7	93.1 ± 39.1	0.002	95.3 ± 36.1	93.0 ± 95.3	0.484
hsCRP (mg/dl)	10.9 ± 29.7	8.9 ± 22.6	0.348	12.8 ± 34.1	9.1 ± 23.1	0.167
NT-proBNP (pmol/L)	1648.3 ± 1568.5	2004.2 ± 1768.5	0.014	1710.0 ± 1641.1	2007.3 ± 1808.3	0.117
HbA1C (%)	6.8 ± 1.3	6.9 ± 1.9	0.707	6.8 ± 1.2	6.8 ± 1.9	0.822
**Discharge medications**						
Aspirin	523 (97.4%)	296 (91.4%)	< 0.001	262 (95.6%)	255 (93.1%)	0.196
Clopidogrel	487 (90.7%)	268 (82.7%)	0.001	238 (86.9%)	227 (82.8%)	0.190
Calcium-channel blocker	108 (20.4%)	87 (26.9%)	0.028	71 (25.9%)	74 (27.0%)	0.771
Beta-blocker	410 (76.9%)	220 (67.9%)	0.023	190 (49.5%)	194 (50.5%)	0.709
ACE inhibitor	242 (45.7%)	130 (40.2%)	0.116	126 (46.3%)	119 (43.6%)	0.521
ARB	193 (36.3%)	104 (32.3%)	0.229	109 (39.8%)	92 (33.7%)	0.140

CAD, coronary artery disease; LVEF, left ventricular ejection fraction; GFR, glomerular filtration rate; CK-MB, creatine kinase MB; TnI, troponin I; HDL, high-density lipoprotein; LDL, low-density lipoprotein; hsCRP, high-sensitivity C-reaction protein; NT-proBNP, N-terminal prohormone of brain natriuretic peptide; HbA1c, hemoglobin A1c; ACE, angiotensin converting enzyme; ARB, angiotensin receptor blocker

### Clinical outcomes in the total population

**Fig 2:**
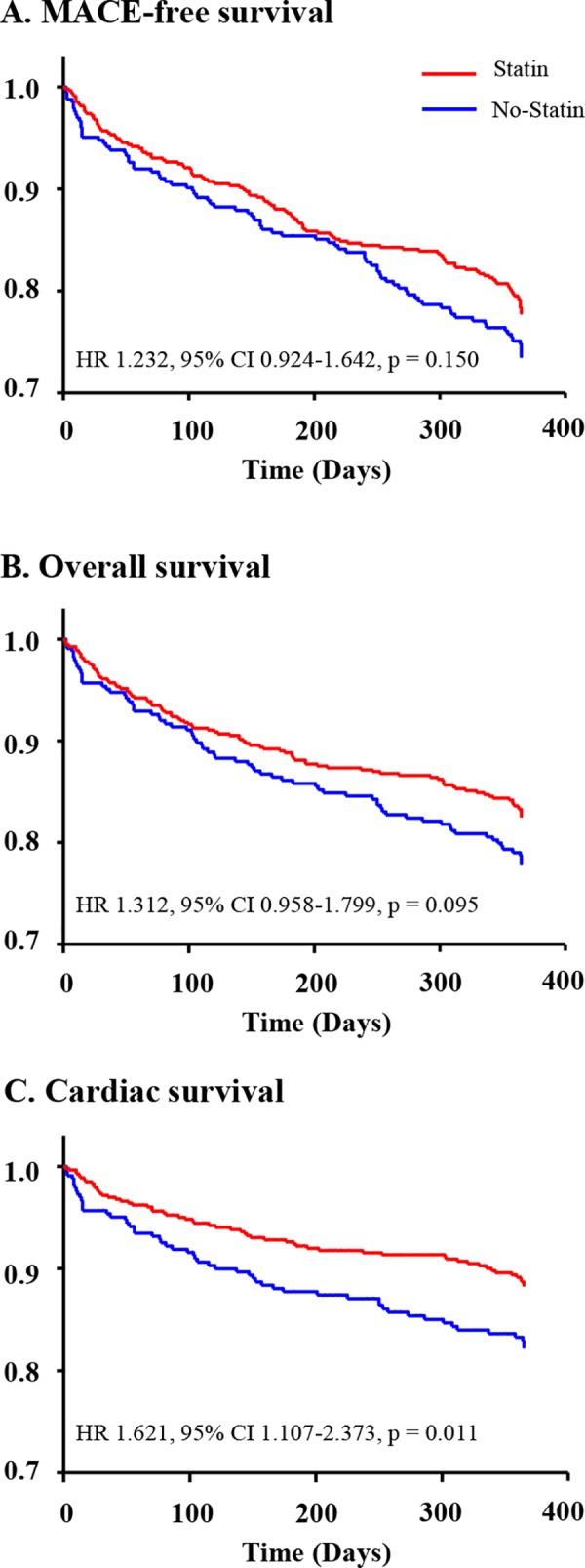
Event-free survival curves in total population. MACE, major adverse cardiac event; HR, hazard ratio; CI, confidence interval.

Clinical outcomes in the total and propensity score-matched population are shown in Table 2. During the 12-month follow-up period, 199 (23.1%) patients experienced MACEs. Of the 199 patients, cardiac death was noted in 113 (56.8%), 31 (15.6%) showed recurrent MI, 49 (24.6%) needed a repeated PCI, and 6 (3.0%) patients underwent CABG. Prevalence of 12-month overall MACEs or all-cause mortality was not significantly different between the two groups (Fig 2A and 2B). No significant differences were observed in rates of recurrent MI, repeated PCI, or CABG between the two groups. However, the cardiac death rate was significantly lower in the statin group compared to the no-statin group (10.8% vs. 17.0%, p = 0.009). Fig 2C shows 12-months cardiac death-free survival. The log-rank test identified that there was a significant association between statin usage and cardiac death-free survival (p = 0.013). Cox-regression analysis revealed that stain usage was not significantly associated with the incidence of 12-month MACEs (S1 Table)

**Table 2:** Clinical outcomes in total and propensity-score matched population.

	Total population		p	Propensity score-matched population		p
	Statin (n = 537)	No-Statin (n = 324)		Statin (n = 274)	No-Statin (n = 274)	
Overall MACEs	115 (21.4%)	84 (25.9%)	0.128	75 (27.4%)	74 (27.0%)	0.924
Cardiac death	58 (10.8%)	55 (17.0%)	0.009	37 (13.5%)	51 (18.6%)	0.103
Recurrent MI	21 (3.9%)	10 (3.1%)	0.529	11 (4.0%)	7 (2.6%)	0.338
Repeated PCI	31 (5.8%)	18 (5.6%)	0.894	24 (8.8%)	15 (5.5%)	0.135
CABG	5 (0.7%)	1 (0.3%)	0.287	3 (1.1%)	1 (0.4%)	0.316
All-cause mortality	94 (17.5%)	72 (22.2%)	0.089	51 (18.6%)	62 (22.6%)	0.245

MACE, major adverse cardiovascular event; MI, myocardial infarction; PCI, percutaneous coronary intervention; CABG, coronary artery bypass grafting.

### Clinical outcomes in the propensity score-matched population

**Fig 3:**
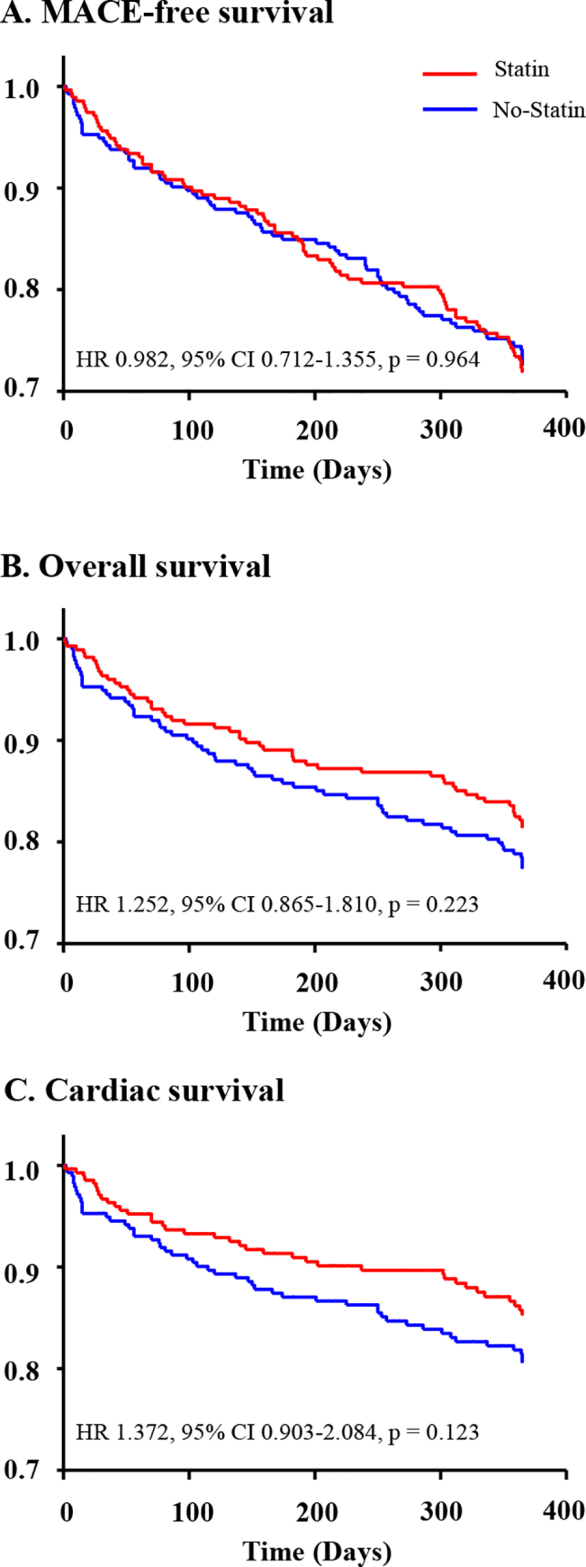
Event-free survival curves in propensity score–matched population. MACE, major adverse cardiac event; HR, hazard ratio; CI, confidence interval.

There were no significant differences in the rates of 12-month overall MACEs or all-cause mortality in the propensity score-matched population (Table 2, Fig 3A and 3B). Furthermore, the prevalence of recurrent MI, repeated PCI, and/or CABG was not significantly different between the two groups. Unlike findings observed in the total population, no significant difference was seen with respect to cardiac death in the propensity score-matched population (Fig 3C). Cox-regression analysis revealed that stain usage did not significantly reduce the incidence of 12-month MACEs or all-cause mortality (Table 3). After adjustment, the left ventricular ejection fraction (LVEF) was the only factor that was independently associated with 12-month MACEs. Higher LVEF has a protective effect on MACEs (HR 0.979, 95% CI 0.962–0.996, p = 0.018).

**Table 3:** Predictors of MACEs in univariate and multivariate Cox regression analyses in propensity-matched population.

Variable	Univariate analysis		Multivariate analysis	
	HR (95% CI)	p	HR (95% CI)	p
Age	1.011 (0.996–1.027)	0.155		
Gender	0.796 (0.577–1.097)	0.163		
Body mass index	0.985 (0.936–1.041)	0.635		
Systolic blood pressure	1.000 (0.995–1.004)	0.860		
Diastolic blood pressure	0.993 (0.984–1.002)	0.107		
Heart rate	0.999 (0.992–1.007)	0.853		
Killip class (more than II)	1.208 (0.865–1.687)	0.267		
Hypertension	1.394 (0.919–2.116)	0.118		
Diabetes mellitus	1.535 (1.092–2.156)	0.014	1.410 (0.930–2.137)	0.106
Dyslipidemia	1.443 (0.847–2.461)	0.178		
Smoking	0.836 (0.587–1.192)	0.323		
Previous CAD	1.246 (0.883–1.765)	0.210		
LVEF	0.972 (0.959–0.986)	< 0.001	0.979 (0.962–0.996)	0.018
Glucose	1.001 (1.000–1.002)	0.128		
Estimated GFR	1.004 (0.986–1.022)	0.692		
Max CK-MB	0.999 (0.997–1.000)	0.091	1.000 (0.998–1.002)	0.615
Max TnI	0.999 (0.996–1.002)	0.475		
Total Cholesterol	1.000 (0.996–1.004)	0.984		
Triglyceride	1.000 (0.999–1.002)	0.613		
HDL-cholesterol	1.003 (0.991–1.016)	0.618		
LDL-cholesterol	0.999 (0.994–1.003)	0.545		
hsCRP	0.999 (0.993–1.006)	0.834		
NT-proBNP	1.000 (1.000–1.000)	0.136		
HbA1C	1.026 (0.867–1.214)	0.766		
Aspirin	1.476 (0.652–3.340)	0.350		
Clopidogrel	1.273 (0.787–2.060)	0.326		
Calcium-channel blocker	0.784 (0.535–1.150)	0.213		
Beta-blocker	1.601 (1.088–2.357)	0.017	1.472 (0.898–2.413)	0.125
ACE inhibitor	0.887 (0.641–1.227)	0.468		
ARB	0.850 (0.605–1.194)	0.349		
Statin	1.018 (0.738–1.404)	0.913		

CAD, coronary artery disease; LVEF, left ventricular ejection fraction; GFR, glomerular filtration rate; CK-MB, cratine kinase MB; TnI, troponin I; HDL, high-density lipoprotein; LDL, low-density lipoprotein; hsCRP, high-sensitivity C-reaction protein; NT-proBNP, N-terminal prohormone of brain natriuretic peptide; HbA1c, hemoglobin A1c; ACE, angiotensin converting enzyme; ARB, angiotensin receptor blocker

## Discussion

This study investigated the clinical effects of statin therapy on 12-month MACEs or all-cause mortality in patients with advanced renal dysfunction who underwent PCI after AMI. To the best of our knowledge, this is the first study to demonstrate a correlation between statins and clinical outcomes in patients with advanced renal dysfunction, using propensity score-matched analysis and the CKD-EPI equation. Our principal findings were: (1) Statin therapy did not reduce 12-month MACEs or all-cause mortality in patients with advanced renal dysfunction (eGFR < 30 mL/min/ 1.73m^2^) who underwent PCI after AMI. (2) Although a significant reduction in cardiac death rate was observed in the statin group of the total population, the statistical significance diminished when propensity score-matching analysis was used. (3) LVEF was an independent predictor of 12-month MACEs in patients with advanced renal dysfunction undergoing PCI after AMI.

Previous studies have proved the benefits of lipid lowering therapies with respect to cardiovascular outcomes, with statins being the mainstay of this therapy [16, 17]. Although a strong body of evidence supports the cardiovascular benefits of statins in the general population, the significance of statin use in patients with renal dysfunction is unclear [5]. Studies performed on patients with early-stage CKD have generally reported beneficial effects of statins on cardiovascular outcomes [8, 18]. However, statins habe not been conclusively shown to reduce the incidence of cardiovascular events in patients with advanced-stage CKD [6, 7]. These results suggest that the benefit of statins on cardiovascular outcomes in patients with renal disease depends up on the severity of renal dysfunction. Few studies have demonstrated the effect of statin therapy on clinical outcomes in patients with CKD after AMI. Moreover, studies that investigated the effect of statins in patients with CKD who underwent PCI are also limited [9–11].

In the present study, we examined the effects of statin therapy on cardiovascular outcomes and all-cause mortality in patients with advanced CKD who underwent PCI for AMI. Statins were not conclusively shown to demonstrate beneficial effects on clinical outcomes, and our finding was similar to results obtained from previous studies [10]. Although the exact mechanisms to explain these results could not be determined, a hypothetical explanation base on speculation could be offered. Several studies suggest that pathogenesis of CVD differs in patients with CKD [12]. Traditional risk factors alone do not explain the higher cardiovascular morbidity and mortality observed in patients with CKD [19]. Other contributory factors such as indlammation, volume overload, anemia, as well as calcium and phosphorus imbalance may have a greater effect on the outcomes of cardiovascular disease in CKD patients [20, 21]. Additionally, malnutrition may worsen cardiovascular outcomes in a patient by aggravating existing inflammation and heart failure, accelerating atherosclerosis, and increasing susceptibility to infection [22]. Statins might be ineffective in patients with advanced CKD but might have some role in patients with mild CKD. A possible explanation could be that unconventional contributory risk factors worsen as the disease progresses. We additionally analyzed the clinical effect of statins by dividing the patient population into quartiles based on eGFR [quartile 1 (eGFR ≥ 90 mL/min/1.73m^2^), quartile 2 (eGFR ≥ 60 to < 90 mL/min/1.73m^2^), quartile 3 (eGFR ≥ 30 to < 60 mL/min/1.73m^2^), and quartile 4 (eGFR < 30 mL/min/1.73m^2^)]. The significant beneficial effect of statins was only observed in patients with eGFR ≥ 90 mL/min/1.73m^2^ (S2 Table).

Prior to performing propensity score-mating, the total cholesterol and LDL cholesterol levels at baseline were noted to be higher in the statin group. Malnutrition could aggravate existing inflammation, thereby contributing to the increased cardiovascular risk in CKD patients. Thus, a higher lipid profile, which indicates a good nutritional status could be a confounding factor while estimating the effect of statin treatment. Therefore, we performed propensity matching using 1:1 propensity score-matching; thus, eliminating confounders including lipid profiles. After propensity matching and elimination of confounders, we could accurately estimate the role of statin treatment in patients with advanced CKD who had been diagnosed with an AMI. We found that statin therapy after MI was not significantly effective for 12-month MACEs or all-cause mortality, and LVEF was the only independent predictor of risk. LVEF represents left ventricle systolic function and is acknowledged to be associated with a mortality risk after MI [23]. In patients with AMI, LVEF may reflect a higher degree of myocardial damage or myocardial stunning [24]. La Rovere et al. [25] have reported that patients with a low LVEF (< 35%) had a relatively higher risk of cardiac morality compared to patients with a high LVEF (> 50%) after an MI. Another recent study has shown that LVEF ≤ 30% effectively predicted cardiac and all-cause mortality in acute MI survivors [26]. In this study, the hazard ratio of LVEF for 12-month MACEs is close to 1, indicating that LVEF might not relevant in daily practice. However, considering 95% CI, it is difficult to assume that LVEF does not possess a good predictive value.

Using multivariate analysis, we failed to establish a significant association between some traditional risk factors and MACEs. Possible suppositions for this phenomenon could be: Our study population comprised only patients with advanced renal dysfunction (eGFR < 30 mL/min/1.73 m^2^). Some studies have failed to reveal a significant association between traditional risk factors and CVD in patients with advanced renal dysfunction [19, 27]. Additionally, as previously reported, the triglyceride to high-density lipoprotein cholesterol ratio failed to predict MACEs after AMI in patients with renal dysfunction [28]. We hypothesized that the above-mentioned conventional and unconventional risk factors have an additive effect in increasing the susceptibility of CKD patients to CVD.

There are some limitations in our study. First, we used the eGFR value instead of directly measuring the glomerular filtration rate (GFR). This could lead to an over- or underestimation of renal function. Second, serum creatinine was measured only once, which could result in an incorrect classification of patients. Although we tried to address this problem, we could not obtain additional data because of the features of the database. We will try to eliminate these drawbacks in our next study using multiple data point collection. Third, we did not have information on the dosages and types of statins administered. Although these factors were considered during analyses, we were unable to obtain complete information. Fourth, participants were not randomized and study was performed on a very specific population. There might be some differences between our study population and members of the general population with renal dysfunction. Of the 33,205 patients who underwent PCI for AMI between November 2005 and July 2012, 1,776 patients with advanced renal dysfunction were selected for this study. The effect of underpowered analysis cannot be denied. However, considering previous studies performed on AMI patients in Korea [29], the prevalence of advanced renal dysfunction in our study is no significantly different. After excluding the ineligible population, 861 patients were found to be eligible for this study. We selected 548 patients using 1:1 propensity score-matching to minimize a selection bias. Finally, we did not assess the long-term clinical outcomes, and did not state the improvement in clinical outcomes after administration of statins. Prospective clinical studies including data that represent improvement of clinical outcomes and studies with a longer follow-up period need to be performed.

There are also some strengths to this study. First, unlike most previous studies which used the Modification of Diet in Renal Disease (MDRD) study equation, we used the recently introduced CKD-EPI equation to calculate the eGFR. This equation uses the same variables (serum creatinine, age, gender, and race) as the MDRD study equation, but applies different coefficients [15]. The CKD-EPI equation reportedly more accurately categorizes mortality risk compared to the MDRD study equation [30]. Second, we performed propensity score-matching analysis. We performed 1:1 matching using the greedy and nearest neighbor algorithm method. In this manner, we could reduce the differences between the two groups and minimize any selection bias. Finally, ours was a population-based study that included a large number of patients.

In conclusion, statin therapy did not significantly reduce the 12-month MACEs or all-cause mortality in patients with advanced renal dysfunction undergoing PCI after AMI. Further longitudinal and comparative studies are required to reliably evaluate the effect of statins in this patient population.

## Appendix

### Institutional review board of participating sites

The Korea Acute Myocardial Infarction Registry (KAMIR) was approved by the institutional review board (IRB) at each participating center (Kyunghee University IRB, Asan Medical Center IRB, Ajou University Hospital IRB, Busan Hanseo Hospital IRB, Busan National University Hospital IRB, Choongbook National University Hospital IRB, Chungnam National University Hospital IRB, Catholic University Hospital IRB, Chonbuk National University Hospital IRB, Chosun University Hospital IRB, Chung-Ang University Hospital IRB, Dankook University Hospital IRB, Daegu Catholic University Medical Center IRB, Dong-A University Medical Center IRB, Daejeon St. Mary's Hospital IRB, Dongguk University Gyeongju Hospital IRB, Gyeongsang National University Hospital IRB, Hallym University Kandong Sacred Heart Hospital IRB, Hallym University Sacred Heart Hospital IRB, Inje University Haeundae Paik hospital IRB, Inje University Sanggye Paik Hospital IRB, Inha University Hospital IRB, Jeju National University Hospital IRB, Kyungpook National University Hospital IRB, Keimyung University Hospital IRB, Korea University Guro Hospital IRB, Konyang University Hospital IRB, Kwangju Christian Hospital IRB, Maryknoll Medical Center IRB, National Health Insurance Corporation Ilsan Hospital IRB, Yeungnam University Hospital IRB, Yonsei University Severance Hospital IRB, Yonsei University Wonju Hospital IRB, Presbyterian Medical Center IRB, Seoul National University Hospital IRB, Seoul National University Bundang Hospital IRB, Soon Chun Hyang University Bucheon Hospital IRB, Soon Chun Hyang University Cheonan Hospital IRB, Samsung Medical Center IRB, Sun General Hospital IRB, Saint Carollo Hospital IRB, Wonkwang University Hospital IRB and Chonnam National University Hospital IRB).

## Supporting information

S1 Table Predictors of MACEs in univariate and multivariate Cox regression analyses before propensity-score matching analysis. (DOCX)

S2 Table Clinical effects of statin therapy on 12-month MACEs according to renal function. (DOCX)

## References

[1] Kwan BC, Kronenberg F, Beddhu S, Cheung AK. Lipoprotein metabolism and lipid management in chronic kidney disease. J Am Soc Nephrol. 2007;18(4):1246–61. doi: 10.1681/ASN.2006091006 . 17360943 10.1681/ASN.2006091006

[2] Yan YL, Qiu B, Wang J, Deng SB, Wu L, Jing XD, et al High-intensity statin therapy in patients with chronic kidney disease: a systematic review and meta-analysis. BMJ Open. 2015;5(5):e006886 doi: 10.1136/bmjopen-2014-006886 ; PubMed Central PMCID: PMCPMC4442158. 25979868 10.1136/bmjopen-2014-006886 PMC4442158

[3] Go AS, Chertow GM, Fan D, McCulloch CE, Hsu CY. Chronic kidney disease and the risks of death, cardiovascular events, and hospitalization. N Engl J Med. 2004;351(13):1296–305. doi: 10.1056/NEJMoa041031 . 15385656 10.1056/NEJMoa041031

[4] Hou W, Lv J, Perkovic V, Yang L, Zhao N, Jardine MJ, et al Effect of statin therapy on cardiovascular and renal outcomes in patients with chronic kidney disease: a systematic review and meta-analysis. Eur Heart J. 2013;34(24):1807–17. doi: 10.1093/eurheartj/eht065 . 23470492 10.1093/eurheartj/eht065

[5] Palmer SC, Craig JC, Navaneethan SD, Tonelli M, Pellegrini F, Strippoli GF. Benefits and harms of statin therapy for persons with chronic kidney disease: a systematic review and meta-analysis. Ann Intern Med. 2012;157(4):263–75. doi: 10.7326/0003-4819-157-4-201208210-00007 ; PubMed Central PMCID: PMCPMC3955032. 22910937 10.7326/0003-4819-157-4-201208210-00007 PMC3955032

[6] Wanner C, Krane V, Marz W, Olschewski M, Mann JF, Ruf G, et al Atorvastatin in patients with type 2 diabetes mellitus undergoing hemodialysis. N Engl J Med. 2005;353(3):238–48. doi: 10.1056/NEJMoa043545 . 16034009 10.1056/NEJMoa043545

[7] Fellstrom BC, Jardine AG, Schmieder RE, Holdaas H, Bannister K, Beutler J, et al Rosuvastatin and cardiovascular events in patients undergoing hemodialysis. N Engl J Med. 2009;360(14):1395–407. doi: 10.1056/NEJMoa0810177 . 19332456 10.1056/NEJMoa0810177

[8] Baigent C, Landray MJ, Reith C, Emberson J, Wheeler DC, Tomson C, et al The effects of lowering LDL cholesterol with simvastatin plus ezetimibe in patients with chronic kidney disease (Study of Heart and Renal Protection): a randomised placebo-controlled trial. Lancet. 2011;377(9784):2181–92. doi: 10.1016/S0140-6736(11)60739-3 ; PubMed Central PMCID: PMCPMC3145073. 21663949 10.1016/S0140-6736(11)60739-3 PMC3145073

[9] Lemos PA, Serruys PW, de Feyter P, Mercado NF, Goedhart D, Saia F, et al Long-term fluvastatin reduces the hazardous effect of renal impairment on four-year atherosclerotic outcomes (a LIPS substudy). Am J Cardiol. 2005;95(4):445–51. doi: 10.1016/j.amjcard.2004.10.008 . 15695126 10.1016/j.amjcard.2004.10.008

[10] Natsuaki M, Furukawa Y, Morimoto T, Sakata R, Kimura T, Investigators CR-KPCRC-. Renal function and effect of statin therapy on cardiovascular outcomes in patients undergoing coronary revascularization (from the CREDO-Kyoto PCI/CABG Registry Cohort-2). Am J Cardiol. 2012;110(11):1568–77. doi: 10.1016/j.amjcard.2012.07.021 . 22935527 10.1016/j.amjcard.2012.07.021

[11] Dasari TW, Cohen DJ, Kleiman NS, Keyes MJ, Yen CH, Hanna EB, et al Statin therapy in patients with chronic kidney disease undergoing percutaneous coronary intervention (from the Evaluation of Drug Eluting Stents and Ischemic Events Registry). Am J Cardiol. 2014;113(4):621–5. doi: 10.1016/j.amjcard.2013.11.006 . 24342762 10.1016/j.amjcard.2013.11.006

[12] Herzog CA, Asinger RW, Berger AK, Charytan DM, Diez J, Hart RG, et al Cardiovascular disease in chronic kidney disease. A clinical update from Kidney Disease: Improving Global Outcomes (KDIGO). Kidney Int. 2011;80(6):572–86. doi: 10.1038/ki.2011.223 . 21750584 10.1038/ki.2011.223

[13] Prichard SS. Impact of dyslipidemia in end-stage renal disease. J Am Soc Nephrol. 2003;14(9 Suppl 4):S315–20. . 12939388 10.1097/01.asn.0000081698.10331.83

[14] Tonelli M, Muntner P, Lloyd A, Manns B, Klarenbach S, Pannu N, et al Association between LDL-C and risk of myocardial infarction in CKD. J Am Soc Nephrol. 2013;24(6):979–86. doi: 10.1681/ASN.2012080870 ; PubMed Central PMCID: PMCPMC3665395. 23687359 10.1681/ASN.2012080870 PMC3665395

[15] Levey AS, Stevens LA, Schmid CH, Zhang YL, Castro AF 3rd, HI Feldman, et al A new equation to estimate glomerular filtration rate. Ann Intern Med. 2009;150(9):604–12. ; PubMed Central PMCID: PMCPMC2763564. 19414839 10.7326/0003-4819-150-9-200905050-00006 PMC2763564

[16] Baigent C, Keech A, Kearney PM, Blackwell L, Buck G, Pollicino C, et al Efficacy and safety of cholesterol-lowering treatment: prospective meta-analysis of data from 90,056 participants in 14 randomised trials of statins. Lancet. 2005;366(9493):1267–78. doi: 10.1016/S0140-6736(05)67394-1 . 16214597 10.1016/S0140-6736(05)67394-1

[17] Prevention of cardiovascular events and death with pravastatin in patients with coronary heart disease and a broad range of initial cholesterol levels. The Long-Term Intervention with Pravastatin in Ischaemic Disease (LIPID) Study Group. N Engl J Med. 1998;339(19):1349–57. doi: 10.1056/NEJM199811053391902 . 9841303 10.1056/NEJM199811053391902

[18] Tonelli M, Isles C, Curhan GC, Tonkin A, Pfeffer MA, Shepherd J, et al Effect of pravastatin on cardiovascular events in people with chronic kidney disease. Circulation. 2004;110(12):1557–63. doi: 10.1161/01.CIR.0000143892.84582.60 . 15364796 10.1161/01.CIR.0000143892.84582.60

[19] Cheung AK, Sarnak MJ, Yan G, Dwyer JT, Heyka RJ, Rocco MV, et al Atherosclerotic cardiovascular disease risks in chronic hemodialysis patients. Kidney Int. 2000;58(1):353–62. doi: 10.1046/j.1523-1755.2000.00173.x . 10886582 10.1046/j.1523-1755.2000.00173.x

[20] Sarnak MJ, Levey AS. Cardiovascular disease and chronic renal disease: a new paradigm. Am J Kidney Dis. 2000;35(4 Suppl 1):S117–31. . 10766010 10.1016/s0272-6386(00)70239-3

[21] Stenvinkel P, Heimburger O, Lindholm B, Kaysen GA, Bergstrom J. Are there two types of malnutrition in chronic renal failure? Evidence for relationships between malnutrition, inflammation and atherosclerosis (MIA syndrome). Nephrol Dial Transplant. 2000;15(7):953–60. . 10862630 10.1093/ndt/15.7.953

[22] Pecoits-Filho R, Lindholm B, Stenvinkel P. The malnutrition, inflammation, and atherosclerosis (MIA) syndrome—the heart of the matter. Nephrol Dial Transplant. 2002;17 Suppl 11:28–31. . 12386254 10.1093/ndt/17.suppl_11.28

[23] Pilote L, Silberberg J, Lisbona R, Sniderman A. Prognosis in patients with low left ventricular ejection fraction after myocardial infarction. Importance of exercise capacity. Circulation. 1989;80(6):1636–41. . 2598426 10.1161/01.cir.80.6.1636

[24] White HD, Norris RM, Brown MA, Brandt PW, Whitlock RM, Wild CJ. Left ventricular end-systolic volume as the major determinant of survival after recovery from myocardial infarction. Circulation. 1987;76(1):44–51. . 3594774 10.1161/01.cir.76.1.44

[25] La Rovere MT, Bigger JT Jr., Marcus FI, Mortara A, Schwartz PJ. Baroreflex sensitivity and heart-rate variability in prediction of total cardiac mortality after myocardial infarction. ATRAMI (Autonomic Tone and Reflexes After Myocardial Infarction) Investigators. Lancet. 1998;351(9101):478–84. . 9482439 10.1016/s0140-6736(97)11144-8

[26] Bauer A, Barthel P, Schneider R, Ulm K, Muller A, Joeinig A, et al Improved Stratification of Autonomic Regulation for risk prediction in post-infarction patients with preserved left ventricular function (ISAR-Risk). Eur Heart J. 2009;30(5):576–83. doi: 10.1093/eurheartj/ehn540 ; PubMed Central PMCID: PMCPMC2649285. 19109245 10.1093/eurheartj/ehn540 PMC2649285

[27] Tsimihodimos V, Mitrogianni Z, Elisaf M. Dyslipidemia associated with chronic kidney disease. Open Cardiovasc Med J. 2011;5:41–8. doi: 10.2174/1874192401105010041 ; PubMed Central PMCID: PMCPMC3106357. 21643500 10.2174/1874192401105010041 PMC3106357

[28] Kim JS, Kim W, Woo JS, Lee TW, Ihm CG, Kim YG, et al The Predictive Role of Serum Triglyceride to High-Density Lipoprotein Cholesterol Ratio According to Renal Function in Patients with Acute Myocardial Infarction. PLoS One. 2016;11(10):e0165484 doi: 10.1371/journal.pone.0165484 ; PubMed Central PMCID: PMCPMC5082929. 27788233 10.1371/journal.pone.0165484 PMC5082929

[29] Bae EH, Lim SY, Cho KH, Choi JS, Kim CS, Park JW, et al GFR and cardiovascular outcomes after acute myocardial infarction: results from the Korea Acute Myocardial Infarction Registry. Am J Kidney Dis. 2012;59(6):795–802. doi: 10.1053/j.ajkd.2012.01.016 . 22445708 10.1053/j.ajkd.2012.01.016

[30] Matsushita K, Mahmoodi BK, Woodward M, Emberson JR, Jafar TH, Jee SH, et al Comparison of risk prediction using the CKD-EPI equation and the MDRD study equation for estimated glomerular filtration rate. JAMA. 2012;307(18):1941–51. doi: 10.1001/jama.2012.3954 ; PubMed Central PMCID: PMCPMC3837430. 22570462 10.1001/jama.2012.3954 PMC3837430

