# Supplementary material for: Effects of statin therapy on clinical outcomes after acute myocardial infarction in patients with advanced renal dysfunction: A propensity score-matched analysis
Source: PLoS One. 2017 Aug 14;12(8):e0183059. doi: 10.1371/journal.pone.0183059 (PMC5555708; doi:10.1371/journal.pone.0183059)
Supplement: S1 Table — (DOCX) [file pone.0183059.s001.docx]

**S Table 1. Predictors of MACEs in univariate and multivariate Cox regression analyses before propensity-score matching analysis**

| **Variable** | **Univariate analysis** | | **Multivariate analysis** | |
| --- | --- | --- | --- | --- |
|  | **HR (95% CI)** | **p** | **HR (95% CI)** | **p** |
| Age | 1.013 (1.000-1.026) | 0.057 | 1.005 (0.988-1.023) | 0.564 |
| Gender | 0.799 (0.605-1.055) | 0.113 |  |  |
| Body mass index | 0.957 (0.916-1.001) | 0.053 | 0.958 (0.904-1.015) | 0.145 |
| Systolic blood pressure | 1.002 (0.998-1.002) | 0.393 |  |  |
| Diastolic blood pressure | 0.998 (0.990-1.005) | 0.528 |  |  |
| Heart rate | 1.004 (0.997-1.010) | 0.254 |  |  |
| Killip class (more than II) | 1.309 (0.983-1.744) | 0.066 | 1.130 (0.766-1.666) | 0.537 |
| Hypertension | 1.225 (0.850-1.764) | 0.276 |  |  |
| Diabetes mellitus | 1.492 (1.112-2.002) | 0.008 | 1.162 (0.759-1.777) | 0.490 |
| Dyslipidemia | 1.310 (0.906-1.985) | 0.151 |  |  |
| Smoking | 0.970 (0.715-1.316) | 0.844 |  |  |
| Previous CAD | 1.350 (1.010-1.804) | 0.043 | 1.445 (0.971-2.150) | 0.069 |
| LVEF | 0.978 (0.967-0.989) | < 0.001 | 0.988 (0.972-1.003) | 0.121 |
| Glucose | 1.001 (1.000-1.002) | 0.099 | 1.001 (0.999-1.002) | 0.228 |
| Estimated GFR | 0.980 (0.985-1.016) | 0.980 |  |  |
| Max CK-MB | 0.999 (0.998-1.001) | 0.364 |  |  |
| Max TnI | 1.000 (0.997-1.002) | 0.857 |  |  |
| Total Cholesterol | 0.999 (0.996-1.002) | 0.455 |  |  |
| Triglyceride | 1.000 (0.998-1.002) | 0.874 |  |  |
| HDL-cholesterol | 1.007 (0.996-1.019) | 0.222 |  |  |
| LDL-cholesterol | 0.998 (0.994-1.001) | 0.175 |  |  |
| hsCRP | 1.002 (0.997-1.007) | 0.534 |  |  |
| NT-proBNP | 1.000 (1.000-1.000) | 0.002 | 1.000 (1.000-1.000) | 0.101 |
| HbA1C | 1.118 (0.970-1.288) | 0.123 |  |  |
| Aspirin | 1.242 (0.612-2.519) | 0.549 |  |  |
| Clopidogrel | 1.054 (0.688-1.614) | 0.810 |  |  |
| Calcium-channel blocker | 0.937 (0.668-1.314) | 0.705 |  |  |
| Beta-blocker | 1.265 (0.907-1.764) | 0.165 |  |  |
| ACE inhibitor | 0.860 (0.647-1.143) | 0.298 |  |  |
| ARB | 0.995 (0.743-1.331) | 0.971 |  |  |
| Statin | 0.832 (0.627-1.101) | 0.197 |  |  |

CAD, coronary artery disease; LVEF, left ventricular ejection fraction; GFR, glomerular filtration rate; CK-MB, cratine kinase MB; TnI, troponin I; HDL, high-density lipoprotein; LDL, low-density lipoprotein; hsCRP, high-sensitivity C-reaction protein; NT-proBNP, N-terminal prohormone of brain natriuretic peptide; HbA1c, hemoglobin A1c; ACE, angiotensin converting enzyme; ARB, angiotensin receptor blocker
