# Supplementary material for: Effects of statin therapy on clinical outcomes after acute myocardial infarction in patients with advanced renal dysfunction: A propensity score-matched analysis
Source: PLoS One. 2017 Aug 14;12(8):e0183059. doi: 10.1371/journal.pone.0183059 (PMC5555708; doi:10.1371/journal.pone.0183059)
Supplement: S2 Table — (DOCX) [file pone.0183059.s002.docx]

**S Table 2. Clinical effects of statin therapy on 12-month MACEs according to renal function**

| eGFR |  |  | Univariate analysis |  | Multivariate analysis* |  |
| --- | --- | --- | --- | --- | --- | --- |
| (mL/min/1.73m^2^) | Statin | No-statin | HR (95% CI) | p | HR (95% CI) | p |
| ≥ 90 | 358/5395 (6.6%) | 126/1469 (8.6%) | 0.766 (0.625-0.939) | 0.010 | 0.760 (0.615-0.939) | 0.011 |
| ≥ 60 to < 90 | 535/7143 (7.5%) | 197/2250 (8.8%) | 0.844 (0.717-0.994) | 0.042 | 0.858 (0.724-1.018) | 0.080 |
| ≥ 30 to < 60 | 296/2632 (11.2%) | 142/1104 (12.9%) | 0.860 (0.704-1.051) | 0.140 | 0.908 (0.734-1.124) | 0.377 |
| < 30 | 115/537 (21.4%) | 84/324 (25.9%) | 0.832 (0.627-1.101) | 0.197 | 0.862 (0.641-1.157) | 0.323 |

eGFR, estimated glomerular filtration rate

*Adjusted by age, gender, body mass index, hypertension, diabetes mellitus, previous coronary artery disease, dyslipidemia
